# Machine-learning based exploration of determinants of gray matter volume in the KORA-MRI study

**DOI:** 10.1038/s41598-020-65040-x

**Published:** 2020-05-20

**Authors:** Franziska Galiè, Susanne Rospleszcz, Daniel Keeser, Ebba Beller, Ben Illigens, Roberto Lorbeer, Sergio Grosu, Sonja Selder, Sigrid Auweter, Christopher L. Schlett, Wolfgang Rathmann, Lars Schwettmann, Karl-Heinz Ladwig, Jakob Linseisen, Annette Peters, Fabian Bamberg, Birgit Ertl-Wagner, Sophia Stoecklein

**Affiliations:** 1 Department of Radiology, University Hospital, LMU Munich, Munich, Germany; 2 Dresden International University, Division of Health Care Sciences, Center for Clinical Research and Management Education, Dresden, Germany; 3 Institute of Epidemiology, Helmholtz Zentrum München, German Research Center for Environmental Health, Neuherberg, Germany; 4 Department of Psychiatry, University Hospital, LMU Munich, Munich, Germany; 5 Munich Center for Neurosciences (MCN), LMU, Munich, Germany; 6 Department of Diagnostic and Interventional Radiology, Rostock University Medical Center, Munich, Germany; 7 Beth Israel Deaconess Medical Center, Boston, Massachusetts, USA; 8 German Centre for Cardiovascular Research (DZHK e.V.), Munich, Germany; 9 Department of Diagnostic and Interventional Radiology, Medical Center - University of Freiburg, Faculty of Medicine, University of Freiburg, Freiburg, Germany; 10 Division of Cardiothoracic Imaging, University Heart Center Freiburg - Bad Krozingen, Bad Krozingen, Germany; 11 German Center for Diabetes Research (DZD), München, Neuherberg Germany; 12 Institute for Biometrics and Epidemiology, German Diabetes Center, Duesseldorf, Germany; 13 Institute of Health Economics and Health Care Management, Helmholtz Zentrum München, German Research Center for Environmental Health, Neuherberg, Germany; 14 Department for Psychosomatic Medicine and Psychotherapy, Klinikum Rechts der Isar, Technische Universität München, Munich, Germany; 15 Chair of Epidemiology, Ludwig-Maximilians-University München, UNIKA-T Augsburg, Augsburg, Germany; 16 Independent Research Group Clinical Epidemiology, Helmholtz Zentrum München, German Research Center for Environmental Health, Neuherberg, Germany; 17 Chair of Epidemiology, Ludwig-Maximilians-University München, Munich, Germany; 18 Department of Radiology, The Hospital for Sick Children, University of Toronto, Toronto, Canada

**Keywords:** Neural ageing, Brain imaging, Magnetic resonance imaging, Epidemiology, Risk factors

## Abstract

To identify the most important factors that impact brain volume, while accounting for potential collinearity, we used a data-driven machine-learning approach. Gray Matter Volume (GMV) was derived from magnetic resonance imaging (3T, FLAIR) and adjusted for intracranial volume (ICV). 93 potential determinants of GMV from the categories sociodemographics, anthropometric measurements, cardio-metabolic variables, lifestyle factors, medication, sleep, and nutrition were obtained from 293 participants from a population-based cohort from Southern Germany. Elastic net regression was used to identify the most important determinants of ICV-adjusted GMV. The four variables age (selected in each of the 1000 splits), glomerular filtration rate (794 splits), diabetes (323 splits) and diabetes duration (122 splits) were identified to be most relevant predictors of GMV adjusted for intracranial volume. The elastic net model showed better performance compared to a constant linear regression (mean squared error = 1.10 vs. 1.59, p < 0.001). These findings are relevant for preventive and therapeutic considerations and for neuroimaging studies, as they suggest to take information on metabolic status and renal function into account as potential confounders.

## Introduction

A plethora of factors such as cardiovascular, metabolic and lifestyle parameters influence gray matter (GM) volume (GMV) of the human brain, with partially overlapping and intercorrelated and partially differential effects.

Among all the potential determinants of GMV, age is probably the best-established factor. There is consensus that a certain decrease of GMV can be considered as a normal function of aging^1,2^. As such, it is common practice to account for age as a cofactor when analyzing volumetric neuroimaging data^3,4^. Previous population-based studies have also identified several other factors that contribute to GMV loss, e.g. reporting negative correlations between GMV and alcohol consumption^5^, (pre-)diabetes^6^, cardiovascular risk factors such as hypertension^7^ and obesity^8^, as well as low levels of physical activity^9^.

These factors usually do not occur isolated but as a combination of multiple risk factors that determine atrophy patterns and rates. Also, diabetes, the metabolic syndrome and alcohol abuse are not dichotomous entities but range within a broad spectrum, defined by multiple parameters such as blood test results, clinical test results, and imaging parameters. Given this broad spectrum of potential risk factors, drawing a clear picture of the most powerful determinants of GMV and atrophy is challenging. While a single factor might be associated with GMV in univariate regression analysis, it is often unclear how important this factor is compared to other parameters that impact GMV.

An exploratory, data-driven approach might be able to not only confirm previously described associated factors influencing GMV, but also provide an estimate about their relative importance and help to identify additional determinants. While traditional confounder-adjusted regression models have limitations when it comes to datasets with a large number of predictors in relation to sample size and potential collinearity of variables, a machine-learning approach may be able to overcome these limitations. For example, the Elastic Net (EN) is a method for variable selection that can select the most important factors from a large pool even in the presence of collinearity^10^.

We analyzed a study sample from a cross-sectional case-control study nested within a prospective population-based cohort without overt cardiovascular disease from the Cooperative Health Research in the Region of Augsburg (KORA) in Southern Germany. Leveraging this rich and unique dataset, we applied multivariate machine-learning algorithms using EN regression to determine potential determinants of intracranial volume (ICV)-adjusted GMV across normoglycemic subjects, subjects with prediabetes and subjects with diabetes.

## Material and Methods

### Study design and participants

**Figure 1:**
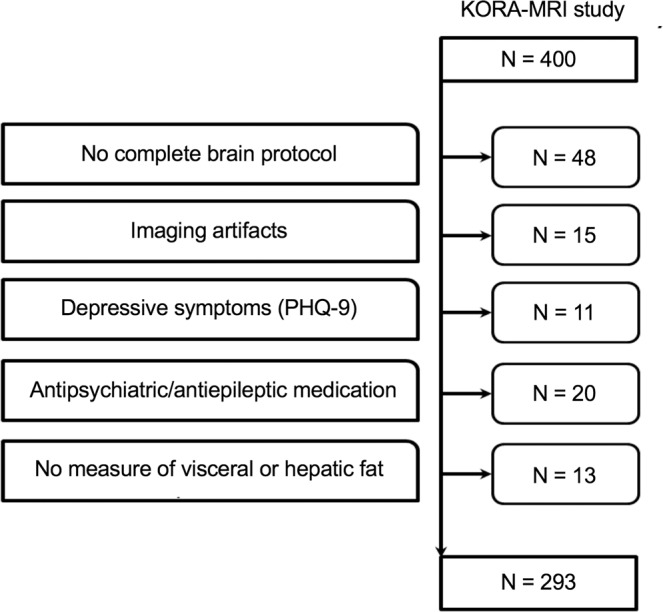
Flowchart of participant selection. PHQ = patient health questionnaire.

We used data from the population-based KORA FF4 study (2013–2014, 2,279 participants). The FF4 study is the second follow-up of subjects that participated in the baseline study (KORA S4, 1999–2001, 4,261 participants). Study design, recruitment of participants and data collection of the KORA studies have been described in detail elsewhere^11^. Nested within the FF4 KORA study, a whole-body magnetic resonance imaging (MRI) sub-study was conducted (n = 400, age 39–73 years) following a case-control design with 54 subjects with diabetes, 103 with prediabetes and 243 with normal glucose metabolism. Inclusion and exclusion criteria for the MRI examination have been described previously^12^. Briefly, subjects with a history of stroke, myocardial infarction and arterial vessel occlusion, type 1 diabetes mellitus or contraindications to MRI were excluded. To avoid bias from neuropsychiatric disease that might effect brain volume we also excluded patients with depressive symptoms and antiepileptic/psychiatric medication. In total, 107 subjects had to be excluded (see flowchart Fig. 1). All subjects underwent MRI within 3 months after their clinical examination at the study center with a median time between the clinical examination and the MRI of 33 days (interquartile range 24–45 days). The majority of MRI exams (67%) were acquired before noon. All study participants gave written informed consent. The study was approved by the ethics committee of the Bavarian Chamber of Physicians and the ethics committee of the Ludwig-Maximilians-University Munich and complies with the Declaration of Helsinki.

### Image acquisition

Fluid-attenuated inversion recovery (T2w FLAIR 3D) sequences of the brain were acquired in supine position using a 3 Tesla MRI scanner (Magnetom Skyra (software syngo MR D13C), Siemens AG, Healthcare Sector, Erlangen, Germany) with a 20-channel phased-array head and neck coil. For all subjects, the same scanner, with no upgrade or software change, was used. The following parameters were used for image acquisition: ST = 0.9 mm, Voxel size inplane = 0.5 ×0.5 mm^2^, field of view (FOV) = 245 ×245 mm, matrix 256 ×256, repetition time (TR) = 5000 ms, echo time (TE) = 389 ms, TI = 1800 ms, flip angle = 120°, and have also previously been described elsewhere^12^.

### Structural brain MRI processing and volumetry

Volumetric GM measurements used for the present study were obtained using a warp-based automated brain volumetric approach based on FLAIR MRI data. T1-weighted images of the brain were not acquired in the KORA-MRI study due to time constraints arising from the whole-body MRI protocol. We have therefore validated FLAIR-based brain volumetry in a recently published study by Beller *et al*. that assessed the feasibility of this approach by correlating the results of FLAIR-based and T1-based volumetry results of 116 atlas regions in 30 healthy individuals^13^.

**Figure 2:**
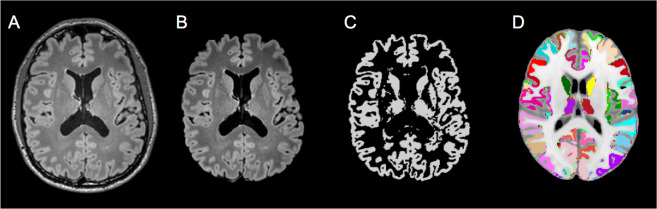
Overview of gray matter volumetry. FLAIR images of each individual (**A**) were reoriented and brain-extracted (**B**). The brain was segmented into gray matter (**C**), white matter and cerebrospinal fluid. Each individuals binarized gray matter map was warped onto the Automatic Anatomical Labeling atlas in MNI space (**D**) using linear and non-linear registration.

Processing of the FLAIR brain MRI data for the present analysis followed the steps described in the above mentioned method paper on the feasibility of FLAIR-based GM volumetry^13^: See Fig. 2 for an overview of the general approach.

Warp-based, automated brain segmentation was applied. Images were pre-processed using FSL 5.0.9 (http://www.fmrib.ox.ac.uk/fsl/index.html) and AFNI (Analyses of Functional Images, http://afni.nimh.nih.gov/afni). Firstly, Fsl2standard (FSL) was used to reorient FLAIR images to match the orientation of the standard template (MNI152). Secondly, the brain was extracted from the re-aligned FLAIR images using the brain extraction tool (BET) implemented in FSL^14^. For brain segmentation into GM, white matter (WM), and cerebrospinal fluid (CSF) FAST (FMRIB’s Automated Segmentation Tool) was used^14^. Individual images were warped onto the Automatic Anatomical Labeling (AAL) atlas^15^ in MNI standard space, applying linear and non-linear registration as implemented in FLIRT and FNIRT^16^. For each individual subject, volumetric data for total GM, WM, CSF, and 116 atlas regions (45 cortical and subcortical regions in each hemisphere, and 26 cerebellar regions^17^) were calculated. To adjust for differences in head size, ratio-corrected brain volumes were calculated by dividing the original uncorrected volume by total intracranial volume (defined as the sum of GM, WM and CSF)^18,19^. See Supplementary Figure 1 for a scatter plot showing the relationship between GMV and ICV. This approach resulted in the final outcome variable for further analysis: ICV-adjusted whole-brain GMV (as a percentage of intracranial volume). The researcher who performed the preprocessing and volumetric analyses (D.K.) was blinded to all potential determinants of ICV-adjusted GMV.

For the analyses of determinants of GMV in the present study, only GM structures that showed a significant correlation between the volumetric results of T1- versus FLAIR-based imaging in the abovementioned validation study^13^ were included. Beller *et al*. considered a Spearman rank correlation coefficient ≥0.597, corresponding to a p-value < 0.0005 between T1- and FLAIR-based GM volume as significant (please see Supplementary Table 1 for an overview of the structures used in the current study)^13^.

### Potential determinants of GMV

To assess the potential influence of variables on ICV-adjusted GMV, 93 predictor variables from the following categories were used: sociodemographics, anthropometric measurements, MRI-derived hepatic and visceral fat, diabetes-related variables, lifestyle factors, somatic symptoms, medication, blood pressure, sleep, laboratory values and nutrition. Sociodemographic variables (e.g. age, family status, education, income), somatic symptoms, medication intake, sleep-related variables (e.g. duration, sleeping problems) were assessed using standardized interviews and questionnaires (please see Supplementary Materials and Supplementary Table 2 for a detailed description of variables and references).

### Descriptive statistics

Continuous predictor variables and ICV-adjusted GMV are presented as either mean and standard deviation (SD) or median with first and third quartile, where appropriate. Categorical predictor variables are presented as counts and percentages. For a description of how missing data were handled, please see Supplemental Materials and Supplementary Table 3.

### Elastic net regression

For outcome ICV-adjusted GMV, a linear EN regression^10^ was calculated using all predictor variables presented in Supplementary Table 4 simultaneously. EN regression is a method for variable selection. The added value of this method compared to traditional linear regression models stems from its ability to provide further information on relative variable importance. This method may be deemed especially helpful for a data-driven analysis of large datasets with multiple, potentially predictive variables with differential impact. While collinearity poses a significant statistical challenge for traditional regression methods, the EN regression selects variables based on their relative importance, even in the presence of collinearity.

EN incorporates the features of both Ridge and least absolute shrinkage and selection operator (LASSO) regression. In Ridge regression (equivalent to a hyperparameter α = 0 in the calculation of the EN), variable coefficients cannot be shrunk to 0, therefore all variables are forced to stay in the model. In LASSO regression (equivalent to a hyperparameter α = 1 in the calculation of the EN), variable coefficients can be shrunk to 0 and therefore variable selection can be performed. By tuning the hyperparameter α, an appropriate amount of variable selection can be achieved.

As there are no established criteria for choosing the best value of the hyperparameter α, it is common to perform the analysis on a grid of sensible α values while minimizing a performance criterion specified a-priori. We minimized the mean squared error (MSE), calculated as MSE = (1/N) * Σ(predicted values – true values)^2^. The root of MSE is on the same scale as the outcome and denotes how many units predicted and actual outcome differ, i.e. a lower root of MSE is indicative of a better ability of the EN to predict the outcome.

We performed all calculations on a grid of α values ranging from α = 0 (Ridge regression) to α = 0.2 in increments of 0.01. Results are presented for α = 0.2, as this α level showed the best tradeoff between parsimonity (at higher α levels and full LASSO, variable selection was too strict to provide an informative model) and prediction performance (at lower α levels, prediction performance as measured by MSE was worse). For an exemplary graph of the influence of changing α values on number of selected splits and β-coefficients see Supplementary Figure 2.

We generated 1000 random data splits. For each data split, the original full data set (N = 293) was divided into 90% (N = 264, respectively) training data and 10% (N = 29, respectively) testing data. Note that due to the different random splitting, the distribution of the original full data set into training and test set is different for each single split of the 1000.

On each of the 1000 training sets, the shrinkage parameter λ was calculated by internal 10-fold cross-validation. This corresponds to a first, inner, cross-validation loop for hyperparameter optimization^20^. With the determined λ value, the linear model with EN penalization was fitted on the full corresponding training data set. The resulting models contain only a subset of the original variables, as coefficients of some variables are shrunk to zero. As a measure of relative variable importance, we report the percentage how often a variable was selected over the 1000 data splits. Furthermore, we report the β-coefficients averaged over 1000 splits for those variables that were selected.

For each of the 1000 data splits, the model derived on the training data set was then applied to the respective held-out test data set, which corresponds to a second, outer, validation loop^20^. By applying the model to the test data set, we obtained predicted values of the outcome GMV. We then calculated the MSE, as a criterion of prediction performance, as well as adjusted R^2^ as a measure of outcome variance explained. We report MSE and adjusted R^2^ averaged over 1000 data splits.

Estimated coefficients from the fitted EN regression are biased towards zero due to the shrinking procedure. Conventional statistical inference, including the calculation of confidence intervals and p-values is therefore not possible. Alternative methods to calculate p-values have not been rigorously established for EN methods and are therefore not reported here.

This also complicates the comparison to unpenalized linear models. Due to the bias in shrunken coefficients^21^, a direct model comparison, including β-coefficients and benchmark metrics, of EN regression and unpenalized regression is not meaningful. For comparison, we therefore used the Null Model, which includes no covariates and always predicts the mean GMV of the sample. It can thus be considered as the minimum bound of predictive performance: Any model that performs worse than this Null Model is not suitable for improving our knowledge about potential predictors of GMV. The Null Model was calculated analogously to the main EN model on all 1000 data splits of training and test data.

To further explore the added value of the variables identified by EN regression, we additionally calculated unpenalized regression models a) including the relevant predictor variables identified by EN regression b) including only age as a predictor variable. For these unpenalized models, 1000 new random data splits of training and testing data were created, again in a 90% to 10% ratio. Unpenalized linear models a) and b) were fitted on the training data. A likelihood-ratio test was computed to test which model fits the data better. Then, models a) and b) were applied to the test data to obtain MSE and R^2^. These metrics can be compared between model a) and b), as both stem from unpenalized regression. We report MSE and adjusted R^2^ averaged over 1000 data splits. The p-values from 1000 Likelihood-Ratio-Tests are displayed by a histogram.

This routine leads to overoptimistic models. Although the data split, and thereby the training and testing data, is different from the one that was used to derive the EN model, the underlying data set is still the same. Hence, the unpenalized regression models using the predictors identified by EN regression are expected to perform better than they would perform on an independent data set. This routine is therefore not recommended to describe the performance of the identified predictors in absolute terms. However, regression models a) and b) are likewise affected by this overoptimism and thus comparison between those two is possible.

As an additional follow-up, correlations between the variables that were identified by EN regression and GMV volume were calculated by permutation tests on 20 000 simulations.

All analyses were conducted with R version 3.4.1. We used package MICE v2.30 for imputation^22^, package glmnet 2.0–13^23^ for the calculation of EN regression and package jmuOutlier 2.2 for permutation testing.

## Results

### Participants

Of 400 subjects from the KORA MRI sub-study, 293 subjects fulfilled the eligibility criteria and were included in the analysis. Baseline characteristics are described in Supplementary Table 4. Briefly, the sample consisted of 59% men, had a mean age of 55.4 ± 9.1 years; 11.9% had diabetes, 23.2% were categorized as subjects with prediabetes and 64.8% had a normal glycemic status.

**Figure 3:**
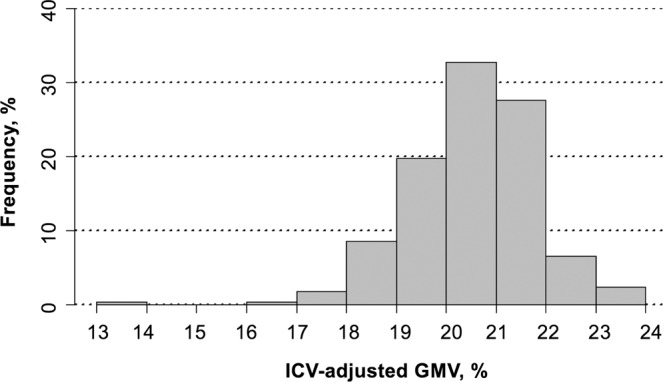
Histogram of ICV-adjusted GMV. ICV = intracranial volume, GMV = gray matter volume.

The mean ICV-adjusted GMV was 20.5% ± 1.3 (see Fig. 3 for the distribution of ICV-adjusted GMV measurements) and mean ICV-adjusted GMV did not show significant differences based on when during the day the MRI was acquired (p = 0.646), see Supplementary Table 5 for details.

### Determinants of ICV-adjusted gray matter volume

**Figure 4:**
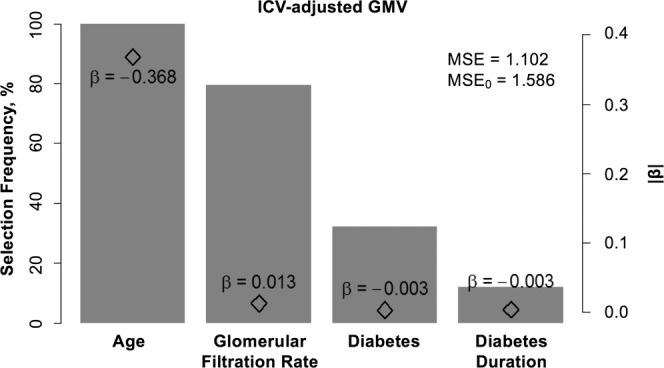
Bar diagram of most important determinants of ICV-adjusted GMV. The left-sided y-axis (bar height) shows the selection frequency in % of 1000 splits where the corresponding variable was selected (α = 0.2), only variables selected in >100 splits are shown. The right-sided axis provides information about the beta coefficient (diamond symbol within bars). ICV = intracranial volume, GMV = gray matter volume, MSE = mean squared error, MSE_0_ = MSE of the Null Model.

The following four variables were identified to be the most predictive (selected in at least 100/1000 splits) of ICV-adjusted GMV: age (selected in each split (1000/1000)), GFR (794 splits), diabetes (323 splits) and diabetes duration (122 splits, see Fig. 4). The MSE of the ICV-adjusted GMV EN regression model (MSE = 1.1015, α = 0.02, mean λ averaged over 1000 splits = 1.08, adjusted R^2^ averaged over 1000 splits = 31%) was lower than the MSE of the Null model (mean ICV-adjusted GMV without adjusting for any cofactors, MSE_0_ = 1.5858), which serves as a proof-of-concept that the EN model provides additional explanatory value of ICV-adjusted GMV. For a detailed overview of selected variables please see Supplementary Table 6A, for performance measures of the EN regression see Supplementary Table 6B. In unpenalized regression models, the model including age, GFR, diabetes and diabetes duration fit the data significantly better than the model including age only (see histogram of p-values in Supplementary Figure 3), provided a slightly better MSE and explained more variance in GMV (see Supplementary Table 7).

While nutrition variables were initially included in the 93 potential determinants of ICV-adjusted GMV, they were not included in the main analyses due to the high number of missing values. To evaluate the influence of nutrition variables, we performed sensitivity analyses only using data from participants where nutrition data was available. In these complete-cases analyses, no nutrition variable was selected for any outcome. Sensitivity analyses were also performed to evaluate a potential influence of prediabetes on ICV-adjusted GMV when excluding all subjects with established type-2 diabetes. However, prediabetes was also not selected as a predictor variable in these sensitivity analyses.

**Figure 5:**
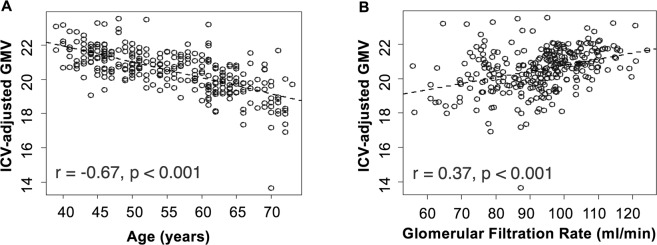
Correlation between ICV-adjusted GMV and (**A**) Age, (**B**) Glomerular Filtration rate. ICV = intracranial volume, GMV = gray matter volume.

**Figure 6:**
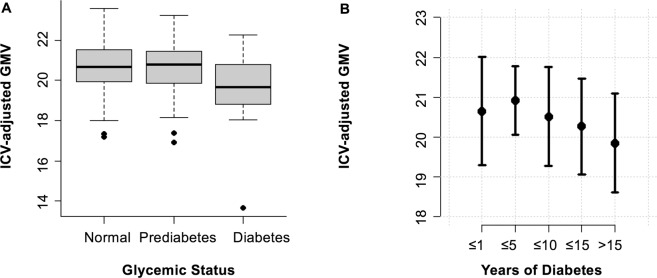
(**A**) Boxplot of ICV-adjusted GMV and glycemic status. (**B**) ICV-adjusted GMV and duration of diabetes. Displayed are mean and standard deviation in the respective groups. ICV = intracranial volume, GMV = gray matter volume.

For ICV-adjusted GMV, age and GFR were selected in the highest number of splits. Figure 5 shows the correlation between these two variables and ICV-adjusted GMV. With increasing age, ICV-adjusted GMV decreases (Pearson’s r = −0.67, 95% Confidence Interval [−0.73, −0.60], permutation-based p-value <0.001). ICV-adjusted GMV was positively correlated with kidney function (Pearson’s r = 0.37, 95%-CI [0.26, 0.46], permutation-based p-value < 0.001). Additionally, diabetes and diabetes duration were selected as predictive variables of ICV-adjusted GMV. Indeed, ICV-adjusted GMV was lower in participants with diabetes compared to normoglycemic subjects (19.7% ± 1.6 vs 20.6% ± 1.2, permutation-based p-value < 0.001), see Fig. 6A. There was no significant difference in ICV-adjusted GMV between subjects with normal glycemic status and those with prediabetes (20.6% ± 1.2 vs 20.7% ± 1.2. p = 1.00). Figure 6B illustrates the decreasing ICV-adjusted GMV with duration of diabetes (Pearson’s r = −0.15, permuation-based p-value = 0.008).

## Discussion

Applying a data-driven, exploratory machine-learning approach, we analyzed a rich dataset from the KORA study and identified age, kidney function, glycemic status and diabetes duration as the most predictive determinants of ICV-adjusted GMV. We could show that EN regression is a feasible approach of leveraging rich population-based datasets to identify and rank predictors from a large pool of variables that impact outcomes such as ICV-adjusted GMV.

Following age, kidney function (indicated by GFR), glycemic status, and diabetes duration were selected as the most predictive variables for ICV-adjusted GMV. Although it has been well known that total GMV declines as a function of age^1,2^ our study extends this knowledge, by putting the impact of age into perspective to the impact of many other potential predictors of GMV. Among these other factors, kidney function was revealed as an important predictor of ICV-adjusted GMV. Changes in GFR have been identified as an early indicator of diabetic nephropathy^24^, a common complication among patients with diabetes. As patients with reduced kidney function often display further risk factors of cerebral changes such as diabetes and hypertension, the exact mechanism of how reduced kidney function – after adjusting for other risk factors – may lead to structural brain changes, has not been fully understood^25^. One hypothesis being discussed is that cerebral small vessel disease leads to white matter hyperintensities among patients with chronic kidney disease^26^. Accordingly, several studies have have found an association between reduced GFR/chronic kidney disease and increased white matter hyperintensities^27,28^. GMV loss has also been found in patients with end stage renal disease, discussing elevated serum urea levels as a potential risk factor^29^.

Our results of diabetes-related variables among the top contributing factors of ICV-adjusted GMV confirm findings from prior studies showing reduced GMV among subjects with diabetes compared to healthy controls (for meta-analysis, see^30^). However, the detailed pathophysiology of the association between diabetes and decreased GMV remains unclear. Potential key factors that have been discussed are detrimental effects of permanently elevated glucose levels and resulting brain insulin resistance. Chronic hyperglycemia has been found to cause accumulation of proinflammatory advanced glycosylation end products which may lead to irreversible tissue damage^31,32^. Brain insulin resistance has been shown to cause impaired synaptogenesis, altered synaptic plasticity, mitochondrial dysfunction and increased tau phosphorylation^33^. Accordingly, the cortical atrophy patterns among patients with diabetes partially resemble those in preclinical Alzheimer disease^34^.

Our findings suggest that other variables, like prediabetes, gender and hypertension may carry relatively less predictive value. Despite the strong impact of diabetes on ICV-adjusted GMV found in this study, prediabetes was not selected as one of its most important predictors, neither in our intial machine-learning approach, nor in a subsequent sensitivity analysis where subjects with type 2 diabetes mellitus were excluded. At first glance, this result might seem to contradict previous reports of reduced GMV in prediabetic subjects^6^. However, the discrepancy may be partially explained by differences between an hypothesis-driven approach and our data-driven, exploratory analysis. While our results do not rule out a potential association between prediabetes and GMV, they merely suggest that other variables from our large pool of potential determinants of GMV are more important for the prediction of ICV-adjusted GMV in our study population without overt cardiovascular disease.

While associations between gender and GMV have been previously reported^3^, gender was not selected as one of the most important determinants of ICV-adjusted GMV in our study, and there was no significant difference of ICV-adjusted GMV between men (20.5±SD 1.3) and women (20.6±SD 1.2, p = 0.316), see Supplementary Figure 4. As our sample size was already limited, we refrained from sex-stratified analyses to maintain statistical power. Analogously, the fact that other previously described determinants of GMV, such as elevated glycated hemoglobin (HbA1c)^35^ and hypertension^7^, were not selected in our analyses, might be rooted in the design of our analysis, which aimed at identifiying only the most powerful among various factors. While hypothesis-driven neuroimaging studies often do not account for metabolic factors such as type 2 diabetes or hypertension^36^, our data-driven analysis included 93 variables. Alleged discrepancies between our study results and previous findings have to be interpreted in consideration of this fundamentally different study design and aim.

Neuroimaging studies investigating volumetric changes in aging and neurodegeneration usually control for age and gender, e.g.^37,38^. Additional metabolic, renal and cardiovascular parameters are mostly not assessed. While our results underline the importance of controlling for age, they also suggest that information on diabetes and renal function should be collected and taken into account when analyzing and interpreting neuroimaging data.

As the KORA-MRI sub-study focused on individuals without overt cardiocerebrovascular disease^39^, the study sample is not entirely representative of the general population. Participants with prediabetes and diabetes are likely to be over-represented in this cohort which may have favored the selection of diabetes-related variables based on the increased power to detect a diabetes-related effect. In addition, we used volumetric data derived from FLAIR images instead of a T1-weighted images of the brain. While the feasibility of FLAIR-based brain volumetry has been assessed in healthy individuals^13^, T1 images are the standard for structural brain analyses. As such, some differences between our findings and previous results may also be a result of sequence-specific differences in brain segmentation. However, by only including GM structures in our ICV-adjusted GMV that have been reported to show significant correlations between T1- and FLAIR-based volumetry results we obtained at a FLAIR-based ICV-adjusted GMV that can serve as a proxy of GMV.

Our statistical machine-learning model identified important covariates by optimizing prediction performance. It is not a formal causal inference model; therefore we cannot claim that the identified predictors are etiologic factors or have causal effects on GMV. To assess causality in a formalized way, other statistical methods have to be used. However, especially for cross-sectional, observational data, these formal methods of causal inference require extensive theoretical reasoning and strong assumptions of the underlying etiologic structures. Particularly in an epidemiological context it is important to consider evidence from a variety of models and study designs to explore causality^40,41^. Therefore, although our model is not causal in the formalized sense, it can still inform causal theory and provide important indications for potential etiologic factors of GMV.

Given the limitations of our study, external validation of our approach using data from other large population-based studies such as UK Biobank^42^ with available brain imaging (including T1-weighted brain images) data, as well as evaluating the influence of different brain parcellation schemes and different machine learning approaches would be beneficial.

## Conclusions

In conclusion, this study shows that EN regression is a feasible machine-learning approach to identify predictors of ICV-adjusted GMV. The results from this data-driven exploratory analysis could potentially generate hypotheses for more focused, hypothesis-driven studies to follow. Additionally, the identification of diabetes-related parameters and GFR as important predictive variables for GMV suggests that these parameters should be taken into account in neuroimaging studies, especially when investigating effects of aging and neurodegeneration.

## Supplementary information

Supplementary Information.

## Data Availability

The informed consent given by KORA study participants does not cover data posting in public databases. However, data are available upon request from KORA/KORA-gen (https://epi.helmholtz-muenchen.de/) by means of a project agreement. Requests should be sent to kora.passt@helmholtz-muenchen.de and are subject to approval by the KORA Board.
