# Supplementary material for: Machine-learning based exploration of determinants of gray matter volume in the KORA-MRI study
Source: Sci Rep. 2020 May 20;10:8363. doi: 10.1038/s41598-020-65040-x (PMC7239887; doi:10.1038/s41598-020-65040-x)
Supplement: Supplementary file 1 — Supplementary Information. [file 41598_2020_65040_MOESM1_ESM.docx]

**Machine-learning based exploration of determinants of gray matter volume in the KORA-MRI study**

Franziska Galiè, MD, MSc^1,2^, Susanne Rospleszcz, MSc^3^, Daniel Keeser^1,4,5^, PhD^1^, Ebba Beller, MD^1,6^, Ben Illigens, MD^2,7^, Roberto Lorbeer, PhD^1,8^, Sergio Grosu, MD^1^, Sonja Selder^1^, Sigrid Auweter, PhD^1^, Christopher L. Schlett, MD, MPH^9,10^, Wolfgang Rathmann, MD, MSPH^11,12^, Lars Schwettmann, PhD^13^, Karl-Heinz Ladwig, PhD^3,14^, Jakob Linseisen, PhD^15,16^, Annette Peters, PhD^3,8,17^, Fabian Bamberg, MD, MPH^9^, Birgit Ertl-Wagner, MD, MHBA^1,18^, Sophia Stoecklein*, MD^1^

**Supplementary Materials and Methods**

**Description of potential determinants of gray matter volume**

Anthropometric variables (e.g. weight, height, waist and hip circumference) were measured in standardized examinations ^1^. Hepatic and visceral fat was derived from magnetic resonance imaging (MRI) measurements as described elsewhere ^2-4^. Diabetes-related measurements were based on laboratory measurements and physician-validated diagnosis of type 2 diabetes mellitus (definition of glycemic status: normal = fasting glucose < 110 mg/dL and 2h glucose < 140 mg/dL, prediabetes = 110 mg/dL ≤ fasting glucose ≤ 125 mg/dL and/or 140 mg/dL ≤ 2-h glucose < 200 mg/dL, diabetes = fasting glucose > 125 mg/dL and/or 2-h glucose ≥ 200 mg/dL), duration of diabetes was calculated based on self-report ^5-7^. Lifestyle factors (alcohol consumption, smoking behavior, physical activity) contain self-reported items (e.g. smoking status) and derived calculations such as pack-years ^8-10^. The blood pressure variables were obtained from standardized measurements (for systolic and diastolic blood pressure, pulse pressure), hypertension was defined as systolic/diastolic blood pressure ≥ 140/90 mmHg and/or intake of antihypertensive medication, given that the participant was aware of being hypertensive ^11^. Laboratory values with a focus on lipids and kidney function were determined by enzymatic assays, for the glomerular filtration rate (GFR) a sex-specific calculation based on serum creatinine according to the Chronic Kidney Disease Epidemiology Collaboration (CKD-EPI) was used ^6,12^. Nutrition variables (daily total energy intake, protein, fat, carbohydrates) were calculated based on one Food Frequency Questionnaire and repeated 24-h Food Lists. Strict quality control implied that individuals with non-available questionnaire or any non-available 24-h Food List were counted as missing.

**Handling of missing data**

As detailed in Figure 1, subjects with missing values in any of the MRI parameters (either GM volume (GMV)-related outcomes or hepatic and visceral fat as predictors) were excluded. Missing values in the predictor variables of interest were infrequent and therefore imputed by single imputation based on predictive mean matching using 5 cases in each match set for continuous variables and logistic regression based for dichotomous variables ^13^, see Supplementary Table 2 for an overview of imputed values. Variables used in the imputation model were all predictor variables of interest as well as all outcome variables. The high number of missing values in the nutrition variables as detailed above would require multiple imputation; however there is no consensus how to pool results from elastic net (EN) analyses obtained from different imputations ^14^; therefore we refrained from imputing the nutrition variables and used those in a complete-cases sensitivity analysis.

**Validation analysis: unpenalized linear regression model**

To validate our results we constructed an unpenalized regression model including the four identified variables (age, glycemic status, diabetes duration, glomerular filtration rate) and compared this model to an unpenalized regression model including only age as a covariate.

To this aim, we repeated the following procedure 1000 times:

- Random split of data into 90% training and 10% testing data. Please note that we used a different splitting compared to the data splitting of the EN model
- Train a linear regression with outcome brain volume and predictors age, glycemic status, diabetes duration, glomerular filtration rate on the training data
- Train a linear regression with outcome brain volume and predictor age on the same training data
- Calculate Likelihood-Ratio Test between the two models: Does the model with multiple predictors fit the data significantly better than the model including only age?
- Predict brain volume on the test data, based on the obtained models
- Calculate mean squared error (MSE) and R^2^ on the test data
- Then average MSE and R^2^ over 1000 splits as measures of predictive performance.

As a measure of model fit, we plotted the distribution of the 1000 p-values obtained from the Likelihood-Ratio Tests. We are aware that this procedure has its drawbacks. Although the data split (and thus the training and testing data) is different from the one that was used to derive the EN model, the underlying data set is still the same. Hence, the unpenalized regression models are expected to perform better than they would perform on an independent data set. However, this affects both the model including multiple predictors and the model including only age, and as we are mainly interested in the comparison between those two, this should not play a major role.

**Supplementary Figures**

**Supplementary Figure 1. Scatter plot showing a positive correlation between ICV and GMV.** This plot shows a positive linear relationship (r = 0.94, p < 0.001) between ICV and GMV, which is the rationale for adjusting GMV for ICV using the ratio-method.

ICV = Intracranial volume, GMV = Gray matter volume.

**Supplementary Figure 2. Exemplary graph of the influence of changing α values on number of selected splits and β-coefficients.** This figure shows how the choice of the α value (on the x-axis) influences the number of selected splits (left y-axis, grey bars) and β-coefficients (right y-axis, red line). Exemplary results for age: the number of selected splits (grey bars) remains constant at N=1000/1000 for all α values between 0 to 0.2 (x-axis), the β-coefficient (red line) decreases with an increasing α.

GMV = Gray matter volume, ICV = Intracranial volume.

**Supplementary Figure 3: Distribution of p-values from 1000 Likelihood-Ratio-Tests between a linear model including age, GFR, diabetes and diabetes duration compared to a model including age only.** The majority of p-values is < 0.05, indicating the larger model fits the data significantly better.

**Supplementary Figure 4. ICV-adjusted GMV by gender.** The boxplots depict the distribution of ICV-adjusted GMV separately for men and women. Mean GMV was not significantly different between men and women: Mean GMV for men was 20.5% with a standard deviation of 1.3 and mean GMV for women was 20.6% with a standard deviation of 1.2, p = 0.3159.

ICV = Intracranial volume, GMV = Gray matter volume.

**Supplementary Tables**

**Supplementary Table 1.** Gray matter structures included in the analysis of gray matter volume. Spearman’s rho and p-values were obtained from a correlation analysis comparing volumetry results based on T1 versus FLAIR images in 30 healthy individuals. Correlation coefficients ≥0.597, corresponding to a p-value <0.0005 (*), were considered significant ^15^.

| Neuroanatomical  structure (GM) | Spearman's rho /  p-value | Brain structure for analysis |
| --- | --- | --- |
| Frontal_Sup_L | **0.623*** | **Frontal** |
|  | 0.0002 |  |
| Frontal_Sup_R | **0.769*** | **Frontal** |
|  | 0.000001 |  |
| Frontal_Sup_Orb_R | **0.807*** | **Frontal** |
|  | 0.0000001 |  |
| Frontal_Mid_L | **0.751*** | **Frontal** |
|  | 0.000002 |  |
| Frontal_Mid_R | **0.689*** | **Frontal** |
|  | 0.00003 |  |
| Frontal_Inf_Oper_L | **0.867*** | **Frontal** |
|  | 0.000000001 |  |
| Frontal_Inf_Oper_R | **0.890*** | **Frontal** |
|  | 0.00000000005 |  |
| Frontal_Inf_Tri_L | **0.741*** | **Frontal** |
|  | 0.000003 |  |
| Frontal_Inf_Tri_R | **0.728*** | **Frontal** |
|  | 0.000005 |  |
| Frontal_Inf_Orb_L | **0.728*** | **Frontal** |
|  | 0.000005 |  |
| Frontal_Inf_Orb_R | **0.793*** | **Frontal** |
|  | 0.0000002 |  |
| Rolandic_Oper_L | **0.779*** | **Frontal** |
|  | 0.0000004 |  |
| Rolandic_Oper_R | **0.873*** | **Frontal** |
|  | 0.0000000003 |  |
| Supp_Motor_Area_L | **0.624*** | **Frontal** |
|  | 0.0002 |  |
| Olfactory_L | **0.611*** | **Frontal** |
|  | 0.0003 |  |
| Frontal_Sup_Medial_L | **0.806*** | **Frontal** |
|  | 0.0000001 |  |
| Frontal_Sup_Medial_R | **0.851*** | **Frontal** |
|  | 0.000000002 |  |
| Frontal_Med_Orb_L | **0.688*** | **Frontal** |
|  | 0.00003 |  |
| Rectus_R | **0.679*** | **Frontal** |
|  | 0.00004 |  |
| Insula_L | **0.599*** | **Insula** |
|  | 0.0005 |  |
| Insula_R | **0.799*** | **Insula** |
|  | 0.0000001 |  |
| Cingulum_Ant_L | **0.880*** | **Cingulate gyrus** |
|  | 0.0000000002 |  |
| Cingulum_Ant_R | **0.872*** | **Cingulate gyrus** |
|  | 0.0000000004 |  |
| Cingulum_Mid_L | **0.925*** | **Cingulate gyrus** |
|  | 0.0000000000003 |  |
| Cingulum_Mid_R | **0.903*** | **Cingulate gyrus** |
|  | 0.00000000001 |  |
| Cingulum_Post_L | **0.910*** | **Cingulate gyrus** |
|  | 0.000000000003 |  |
| Cingulum_Post_R | **0.907*** | **Cingulate gyrus** |
|  | 0.00000000001 |  |
| Hippocampus_L | **0.651*** | **Hippocampus** |
|  | 0.0001 |  |
| Hippocampus_R | **0.709*** | **Hippocampus** |
|  | 0.00001 |  |
| Occipital_Sup_R | **0.754*** | **Occipital** |
|  | 0.000002 |  |
| Occipital_Mid_L | **0.598*** | **Occipital** |
|  | 0.0005 |  |
| Occipital_Mid_R | **0.743*** | **Occipital** |
|  | 0.000003 |  |
| Fusiform_R | **0.624*** | **Temporal** |
|  | 0.0002 |  |
| Parietal_Sup_R | **0.723*** | **Parietal** |
|  | 0.00001 |  |
| Parietal_Inf_L | **0.713*** | **Parietal** |
|  | 0.00001 |  |
| Parietal_Inf_R | **0.920*** | **Parietal** |
|  | 0.000000000001 |  |
| SupraMarginal_L | **0.860*** | **Parietal** |
|  | 0.000000001 |  |
| SupraMarginal_R | **0.849*** | **Parietal** |
|  | 0.000000003 |  |
| Angular_L | **0.874*** | **Parietal** |
|  | 0.0000000003 |  |
| Angular_R | **0.873*** | **Parietal** |
|  | 0.0000000003 |  |
| Precuneus_L | **0.621*** | **Parietal** |
|  | 0.0002 |  |
| Precuneus_R | **0.819*** | **Parietal** |
|  | 0.00000003 |  |
| Pallidum_L | **0.701*** | **Pallidum** |
|  | 0.00002 |  |
| Heschl_L | **0.778*** | **Temporal** |
|  | 0.0000004 |  |
| Heschl_R | **0.781*** | **Temporal** |
|  | 0.0000004 |  |
| Temporal_Sup_L | **0.844*** | **Temporal** |
|  | 0.000000005 |  |
| Temporal_Sup_R | **0.707*** | **Temporal** |
|  | 0.00001 |  |
| Temporal_Pole_Sup_L | **0.701*** | **Temporal** |
|  | 0.00002 |  |
| Temporal_Pole_Sup_R | **0.863*** | **Temporal** |
|  | 0.000000001 |  |
| Temporal_Mid_L | **0.723*** | **Temporal** |
|  | 0.000006 |  |
| Temporal_Mid_R | **0.663*** | **Temporal** |
|  | 0.00007 |  |
| Temporal_Pole_Mid_R | **0.842** | **Temporal** |
|  | 0.00000001 |  |
| Temporal_Inf_R | **0.674*** | **Temporal** |
|  | 0.00004 |  |
| Cerebelum_3_R | **0.652*** | **Cerebellum** |
|  | 0.0001 |  |
| Cerebelum_10_L | **0.819*** | **Cerebellum** |
|  | 0.00000003 |  |
| Cerebelum_10_R | **0.764*** | **Cerebellum** |
|  | 0.000001 |  |
| Vermis_1_2 | **0.778*** | **Vermis** |
|  | 0.0000004 |  |
| Vermis_3 | **0.691*** | **Vermis** |
|  | 0.00002 |  |
| Vermis_4_5 | **0.656*** | **Vermis** |
|  | 0.0001 |  |
| Vermis_10 | **0.768*** | **Vermis** |
|  | 0.000001 |  |

**Supplementary Table 2.** Detailed overview of variables within each categories, data acquisition and further references.

| **Variable** | **Description** |
| --- | --- |
| **Sociodemographics** | **Further reference: ^5,16-18^** |
| Age, years | Self-reported in standardized interview |
| Family status | Self-reported in standardized interview |
| Schooling | Self-reported in standardized interview |
| Schooling, years | calculated based on self-report in standardized interview |
| Highest professional degree | Self-reported in standardized interview |
| Per-capita income, Euro | calculated based on self-report in standardized interview |
| Equivalence income, Euro | calculated based on per-capita income, weighted according to number and age of all household members. Weights are derived according to cost of living, following Bundessozialhilfegesetz (BSHG) |
| Social stratum, Helmert scale | numeric score based on schooling, degree, job position and equivalence income |
| **Anthropometric measurements** | **Further reference: ^1^** |
| Weight, kg | measured in standardized examination by calibrated steelyards or digital scales (SECA 635 or SECA 877 or SECA measuring station 285, Seca GmbH & Co, KG, Hamburg, Germany) |
| Height, cm | measured in standardized examination by calibrated levelling bar (SECA 242, Seca GmbH & Co, KG, Hamburg, Germany) |
| BMI, kg/m2 | calculated as weight in kg divided by squared height in m |
| Waist circumference, cm | measured in standardized examination with an inelastic tape at the level midway between the lower rib margin and the iliac crest |
| Hip circumference, cm | measured in standardized examination with an inelastic tape at the level of maximal gluteal protrusion |
| Waist-To-Hip Ratio | calculated as waist circumference in cm divided by hip circumference in cm |
| right-handed | Self-reported in standardized interview |
| **Other metabolic measurements** | **Further reference: ^2-4^** |
| Hepatic Fat, % | MRI measurement: proton density fat fraction by multiecho single-voxel 1H spectroscopy |
| Visceral Fat, l | MRI measurement: calculated semiautomatically from volume-interpolated three-dimensional in/opposed-phase volumetric interpolated Dixon sequence from femoral head to the diaphragm |
| **Diabetes related measurements** | **Further reference: ^5-7^** |
| Glycemic Status | determined as either established type-2 diabetes (validated by physician) or after OGTT according to WHO criteria. OGTT was based on 300ml of liquid containing 75g of carbohydrates. |
| normal | fasting glucose < 110 mg/dL and 2h glucose < 140 mg/dL |
| prediabetes | 110 mg/dL <= fasting glucose <= 125 mg/dL and/or 140 mg/dL <= 2-h glucose <= 200 mg/dL |
| diabetes | fasting glucose > 140 mg/dL and/or 2-h glucose > 200 mg/dL |
| Duration of diabetes, years | calculated based on self report |
| Fasting glucose, mg/dL (Serum) | UV test using enzymatic reference method with hexokinase (Vista, Siemens or Cobas, Roche) |
| Fasting insulin, mg/dL (Serum) | Elecsys Insulin immunoassay with two monoclonal antibodies (Vista, Siemens or Cobas, Roche) |
| HbA1c, % (hemolyzed whole blood) | cation-exchange high performance liquid chromatographic, photometric assay (VARIANT II TURBO Hemoglobon Testing System, Bio-Rad Laboratories Inc, Hercules, US) |
| **Lifestyle factors** | **Further reference: ^8-10^** |
| Alcohol consumption, categorical or g/day | calculated based on self-reported amount and type of alcoholic beverages consumed |
| Smoking | Self-reported in standardized interview |
| Packyears | calculated based on self-reported number of cigarettes smoked |
| Physically active | Self-reported in standardized interview |
| Physical activity | calculated based on self-report in standardized interview |
| **Somatic Symptoms** | **Further reference: ^19^** |
| Angina Pectoris | determined based on self-reported symptoms in standardized interview |
| Sf-12 Somatic Scale | determined based on standardized questionnaire |
| **Medication intake** | **Further reference: ^20^**  based on standardized interview. Participants were asked to bring packages of every medication that they had taken in the 7 days before the interview. Additionally, medication intake was assessed by interview. |
| Antidiabetic | ATC Codes A10 |
| Antihypertensive | compounds from ATC Codes C02, C03, C07, C08, C09 when German guidelines classify the compound as anti-hypertensive |
| Anticoagulant | ATC Codes B01AA, B01AB, B01AE, B01AF, B01AX |
| Antiplatelet | ATC Codes B01AC |
| Thyroidal | ATC Codes H03 (but not H03PB, H03BP, H03CA) |
| NSAID | ATC Codes N02B or M01A (but not M01AX), R05XA, N02AA59, N02AA69, N02AX62 |
| ASS 100/300 | ATC Codes B01AC06 |
| **Blood pressure** | **Further reference: ^11^** |
| Systolic BP, mmHg | 3 measurements with an oscillometric digital device (OMRON HEM-705CP). Average of 2nd and 3rd measurements. |
| Diastolic BP, mmHg | 3 measurements with an oscillometric digital device (OMRON HEM-705CP). Average of 2nd and 3rd measurements. |
| Pulse Pressure | 3 measurements with an oscillometric digital device (OMRON HEM-705CP). Average of 2nd and 3rd measurements. |
| Hypertension | defined as systolic/diastolic blood pressure above 140/90 mmHg or intake of antihypertensive medication, given that the participant was aware of being hypertensive. |
| Control and awareness of hypertension | based on blood pressure measurements as detailed above, self-reported diagnosis of hypertension by a physician and intake of antihypertensive medication |
| **Sleep** | **Further reference: ^21^** |
| Sleep, h/day | Self-reported in standardized interview |
| Problems falling asleep | Self-reported in standardized interview |
| Problems keeping asleep | Self-reported in standardized interview |
| Feeling tired and exhausted because of sleep problems | Self-reported in standardized interview |
| **Laboratory values** | **Further reference: ^6,22^** |
| Glomerular Filtration Rate | sex-specific calculation based on serum creatinine according to CKD-EPI |
| Total cholesterol, mg/dL (Serum) | Enzymatic, colorimetric CHOL Flex assay (Vista, Siemens or Cobas, Roche) |
| HDL cholesterol, mg/dL (Serum) | Enzymatic, colorimetric LDLC Flex assay (Vista, Siemens or Cobas, Roche) |
| LDL cholesterol, mg/dL (Serum) | Enzymatic, colorimetric HDLC Flex assay (Vista, Siemens or Cobas, Roche) |
| Triglycerides, mg/dL (Serum) | Enzymatic, colorimetric TRIG Flex assay (Vista, Siemens or Cobas, Roche) |
| Uric Acid, mg/dL (Serum) | Enzymatic colorimetric UA Flex assay (Vista, Siemens or Cobas, Roche) |
| Creatinine, mg/dL (Serum) | Kinetic colorimetric CREJ assay based on Jaffé method |
| **Nutrition** |  |
| Total energy intake, kcal/day | calculated based on Food Frequency Questionnaire and 24-h Food List |
| Protein, mg/day | calculated based on Food Frequency Questionnaire and 24-h Food List |
| Fat, mg/day | calculated based on Food Frequency Questionnaire and 24-h Food List |
| Carbohydrates, mg/day | calculated based on Food Frequency Questionnaire and 24-h Food List |

*please not that although the reference might not pertain to KORA FF4 but to one of the other KORA surveys, the described procedure was also applicable in FF4

**Supplementary Table 3.** Number of imputed values per variable for variables with missing data.

| **Variable** | **Number of missing values that had to be imputed** |
| --- | --- |
| Per Capita income | 15 |
| Equivalence income | 15 |
| Packyears | 5 |
| Diabetes duration | 3 |
| Fasting Serum Glucose | 1 |
| SF-12 Somatic Scale | 11 |
| Fasting Serum Insulin | 1 |
| HbA1c | 1 |
| Angina Pectoris | 2 |

HbA1c = Hemoglobin A1c, SF-12 = Short form 12.

**Supplementary Table 4.** Baseline characteristics of the KORA study sample used for further analysis.

|  | N = 293 |
| --- | --- |
| **Sociodemographics** |  |
| Age, years | 55.4 ± 9.1 |
| Gender |  |
| male | 173 (59.0%) |
| female | 120 (41.0%) |
| Family status |  |
| married, living with partner | 219 (74.7%) |
| unmarried, living alone | 24 (8.2%) |
| unmarried, living with partner | 14 (4.8%) |
| married, not living with partner | 4 (1.4%) |
| divorced | 21 (7.2%) |
| widowed | 11 (3.8%) |
| Schooling |  |
| lower secondary school | 129 (44.0%) |
| secondary school | 69 (23.5%) |
| higher secondary school | 95 (32.4%) |
| Schooling, years | 12.3 ± 2.7 |
| Highest professional degree |  |
| no degree | 10 (3.4%) |
| apprenticeship | 150 (51.2%) |
| vocational/technician/master craftsman degree | 70 (23.9%) |
| engineering/polytechnic degree | 4 (1.4%) |
| university degree | 59 (20.1%) |
| Per-capita income, Euro | 1402.4 ± 695.3 |
| Equivalence income, Euro | 1545.6 ± 705.5 |
| Social stratum, Helmert scale | 15.9 ± 5.1 |
| **Anthropometric measurements** | |
| Weight, kg | 82.5 ± 16.0 |
| Height, cm | 172.4 ± 9.6 |
| BMI, kg/m2 | 27.7 ± 4.7 |
| Waist circumference, cm | 97.4 ± 13.6 |
| Hip circumference, cm | 106.6 ± 8.9 |
| Waist-To-Hip Ratio | 0.9 ± 0.1 |
| right-handed | 269 (91.8%) |
| **MRI-derived metabolic measurements** | |
| Hepatic Fat, % | 8.1 ± 7.3 |
| Visceral Fat, l | 4.4 ± 2.6 |
| **Diabetes related measurements** | |
| Glycemic Status |  |
| normal | 190 (64.8%) |
| prediabetes | 68 (23.2%) |
| diabetes | 35 (11.9%) |
| Duration of diabetes, years (median [1^st^ quartile, 3^rd^ quartile]) | 6.0 [0.0, 7.0] |
| Fasting serum glucose, mg/dL | 103.0 ± 21.3 |
| Fasting serum insulin, mg/dL | 10.6 ± 6.6 |
| HbA1c, % | 5.5 ± 0.7 |
| **Lifestyle factors** |  |
| Alcohol |  |
| no consumption | 71 (24.2%) |
| < 20 g/day | 114 (38.9%) |
| < 40 g/day | 59 (20.1%) |
| > 40 g/day | 49 (16.7%) |
| Alcohol consumption, g/day | 17.8 ± 21.6 |
| spirits, g/day | 0.5 ± 1.7 |
| wine, g/day | 5.6 ± 10.4 |
| beer, g/day | 11.6 ± 18.8 |
| Smoking |  |
| neversmoker | 112 (38.2%) |
| ex-smoker | 124 (42.3%) |
| smoker | 57 (19.5%) |
| Pack years (median [1^st^ quartile, 3^rd^ quartile]) | 4.0 [0.0, 21.6] |
| Physically active | 184 (62.8%) |
| Physical activity |  |
| no | 68 (23.2%) |
| sporadic | 41 (14.0%) |
| regularly, around 1 h/week | 96 (32.8%) |
| regularly, 2h/week | 88 (30.0%) |
| **Somatic Symptoms** |  |
| Angina Pectoris | 13 (4.4%) |
| Sf-12 Somatic Scale | 50.2 ± 6.8 |
| **Medication** |  |
| Antidiabetic | 19 (6.5%) |
| Antihypertensive | 64 (21.8%) |
| Anticoagulant | 6 (2.0%) |
| Antiplatelet drugs | 8 (2.7%) |
| Thyroidal | 49 (16.7%) |
| NSAID | 5 (1.7%) |
| ASS 100 or 300 mg | 7 (2.4%) |
| **Blood pressure** |  |
| Systolic BP, mmHg | 120.6 ± 16.5 |
| Diastolic BP, mmHg | 75.5 ± 10.3 |
| Pulse Pressure | 70.8 ± 10.0 |
| Hypertension | 94 (32.1%) |
| Control and awareness of hypertension | |
| no hypertension | 199 (67.9%) |
| controlled hypertension | 50 (17.1%) |
| uncontrolled hypertension | 14 (4.8%) |
| hypertension, untreated | 17 (5.8%) |
| hypertension, unknown | 13 (4.4%) |
| **Sleep** |  |
| Sleep, h/day | 7.1 ± 1.0 |
| Problems falling asleep |  |
| never | 196 (66.9%) |
| sometimes | 74 (25.3%) |
| often | 23 (7.8%) |
| Problems keeping asleep |  |
| never | 134 (45.7%) |
| sometimes | 106 (36.2%) |
| often | 53 (18.1%) |
| Feeling tired and exhausted because of sleep problems |  |
| never | 197 (67.2%) |
| sometimes | 85 (29.0%) |
| often | 11 (3.8%) |
| **Laboratory values** |  |
| Glomerular Filtration Rate | 92.9 ± 13.0 |
| Total cholesterol, mg/dL | 217.2 ± 37.1 |
| HDL cholesterol, mg/dL | 61.7 ± 17.9 |
| LDL cholesterol, mg/dL | 140.0 ± 32.9 |
| Triglycerides, mg/dL | 127.7 ± 80.2 |
| Uric Acid, mg/dL | 5.6 ± 1.5 |
| Creatinine, mg/dL | 0.9 ± 0.2 |
| **Nutrition** |  |
|  | N = 230 |
| Total energy intake, kcal/day | 1846.1 ± 417.2 |
| Protein, mg/day | 70088.8 ± 15230.9 |
| Fat, mg/day | 77199.8 ± 16732.9 |
| Carbohydrates, mg/day | 193401.4 ± 49938.6 |

Continuous variables are displayed as mean ± standard deviation unless otherwise indicated, categorical variables as counts and percentages.

**Supplementary Table 5.** Time of MRI acquisition and ICV-adjusted GMV.

| **Start of MRI examination** | **N*** | **% of sample** | **ICV-adjusted GMV**  **(mean ± SD)** |
| --- | --- | --- | --- |
| 8:00 am - 9:00 am | N = 57 | 19.6 | 20.33 ± 1.25 |
| 9:00 am - 10:00 am | N = 55 | 18.9 | 20.62 ± 1.37 |
| 10:00 am - 11:00 am | N = 47 | 16.2 | 20.78 ± 1.12 |
| 11:00 am - 12:00 am | N = 35 | 12.0 | 20.51 ± 1.22 |
| 12:00 am - 1:00 pm | N = 40 | 13.7 | 20.59 ± 1.02 |
| 1:00 pm - 2:00 pm | N = 30 | 10.3 | 20.49 ± 1.16 |
| 2:00 pm - 3:00 pm | N = 20 | 6.9 | 20.26 ± 1.03 |
| 3:00 pm - 4:00 pm | N = 7 | 2.4 | 20.16 ± 3.13 |
| p-value |  |  | 0.646 |

*Information was available for N = 291 individuals (For N=2, this information is missing).

GMV = Gray matter volume, ICV = Intracranial volume.

**Supplementary Table 6A.** Results of EN regression for ICV-adjusted GMV.

|  | **Variable selected in …of 1000 splits** | **Average of the b-coefficient** |
| --- | --- | --- |
| (Intercept) | 1000 | 20.52872522 |
| Age | 1000 | -0.368049201 |
| GFR | 794 | 0.012531433 |
| Diabetes | 323 | -0.003088488 |
| Diabetes duration | 122 | -0.003365875 |
| Diabetes Medication | 73 | -0.000987547 |
| ASS 100/300 | 49 | -0.000507898 |
| Antiplatelet Medication | 13 | -8.00844E-05 |
| Weight | 7 | 3.46395E-05 |
| BMI | 5 | 4.55193E-05 |
| Physical Activity | 5 | -4.7837E-05 |
| Problems Falling asleep | 4 | 2.30483E-05 |
| Family status: living with partner, unmarried | 3 | 2.14249E-05 |
| Hip Circumference | 3 | 1.20593E-05 |
| Antihypertensive Medication | 3 | -1.12569E-05 |
| Family Status: living alone | 1 | 7.74182E-06 |
| Sleep, duration | 1 | -2.49656E-06 |

GFR = Glomerular filtration rate

**Supplementary Table 6B.** Performance measures and technical properties of EN regression for ICV-adjusted GMV.

| **Model performance** | |
| --- | --- |
| MSE | 1.10150571 |
| MSE of Null Model | 1.58579542 |
| **Technical properties** | |
| α | 0.2 |
| λ.1se | 1.08826323 |

MSE = mean squared error. The Null Model includes no covariates and always predicts mean GMV. α was determined on a grid of values from 0 to 1 and based on the combination of predictive performance and parsimonity. λ.1se was determined by internal 10-fold cross validation.

**Supplementary Table 7.** Performance measure of unpenalized regression models.

|  | **value, averaged over 1000 splits** |
| --- | --- |
| MSE of model with 4 predictors | 0.8872972 |
| MSE of model with only age | 0.8950096 |
| MSE of Null Model | 1.614519 |
| adjusted R^2^ of model with 4 predictors | 0.4611711 |
| adjusted R^2^ of model with only age | 0.4450525 |
| adjusted R^2^ of Null Model | 0 |

MSE = mean squared error. We compare an unpenalized regression model including the four identified variables (age, glycemic status, diabetes duration, glomerular filtration rate) and compared this model to an unpenalized regression model including only age as a covariate and the Null Model. R2 values show that it explains more variability in the data. MSE shows that the model with 4 predictors has slightly better predictive abilities.

**References of Supplementary Materials**

1 Lorbeer, R. *et al.* Correlation of MRI-derived adipose tissue measurements and anthropometric markers with prevalent hypertension in the community. *Journal of hypertension*, doi:10.1097/hjh.0000000000001741 (2018).

2 Bamberg, F. *et al.* Subclinical Disease Burden as Assessed by Whole-Body MRI in Subjects With Prediabetes, Subjects With Diabetes, and Normal Control Subjects From the General Population: The KORA-MRI Study. *Diabetes* **66**, 158-169, doi:10.2337/db16-0630 (2017).

3 Storz, C. *et al.* Phenotypic Multiorgan Involvement of Subclinical Disease as Quantified by Magnetic Resonance Imaging in Subjects With Prediabetes, Diabetes, and Normal Glucose Tolerance. *Investigative radiology* **53**, 357-364, doi:10.1097/rli.0000000000000451 (2018).

4 Hetterich, H. *et al.* Feasibility of a three-step magnetic resonance imaging approach for the assessment of hepatic steatosis in an asymptomatic study population. *European radiology* **26**, 1895-1904, doi:10.1007/s00330-015-3966-y (2016).

5 Rathmann, W. *et al.* High prevalence of undiagnosed diabetes mellitus in Southern Germany: target populations for efficient screening. The KORA survey 2000. *Diabetologia* **46**, 182-189, doi:10.1007/s00125-002-1025-0 (2003).

6 Laxy, M. *et al.* Quality of Diabetes Care in Germany Improved from 2000 to 2007 to 2014, but Improvements Diminished since 2007. Evidence from the Population-Based KORA Studies. *PLoS One* **11**, e0164704, doi:10.1371/journal.pone.0164704 (2016).

7 Kowall, B. *et al.* Perceived risk of diabetes seriously underestimates actual diabetes risk: The KORA FF4 study. *PloS one* **12**, e0171152, doi:10.1371/journal.pone.0171152 (2017).

8 Meisinger, C., Lowel, H., Thorand, B. & Doring, A. Leisure time physical activity and the risk of type 2 diabetes in men and women from the general population. The MONICA/KORA Augsburg Cohort Study. *Diabetologia* **48**, 27-34, doi:10.1007/s00125-004-1604-3 (2005).

9 Schneider, B. *et al.* The effect of risky alcohol use and smoking on suicide risk: findings from the German MONICA/KORA-Augsburg Cohort Study. *Social psychiatry and psychiatric epidemiology* **46**, 1127-1132, doi:10.1007/s00127-010-0287-y (2011).

10 Zeilinger, S. *et al.* Tobacco smoking leads to extensive genome-wide changes in DNA methylation. *PloS one* **8**, e63812, doi:10.1371/journal.pone.0063812 (2013).

11 Lorbeer, R. *et al.* Association between MRI-derived hepatic fat fraction and blood pressure in participants without history of cardiovascular disease. *Journal of hypertension* **35**, 737-744, doi:10.1097/hjh.0000000000001245 (2017).

12 Inker, L. A. *et al.* Estimating glomerular filtration rate from serum creatinine and cystatin C. *The New England journal of medicine* **367**, 20-29, doi:10.1056/NEJMoa1114248 (2012).

13 Morris, T. P., White, I. R. & Royston, P. Tuning multiple imputation by predictive mean matching and local residual draws. *BMC medical research methodology* **14**, 75, doi:10.1186/1471-2288-14-75 (2014).

14 Wan, Y., Datta, S., Conklin, D. J. & Kong, M. Variable selection models based on multiple imputation with an application for predicting median effective dose and maximum effect. *Journal of statistical computation and simulation* **85**, 1902-1916, doi:10.1080/00949655.2014.907801 (2015).

15 Beller, E. *et al.* T1-MPRAGE and T2-FLAIR segmentation of cortical and subcortical brain regions-an MRI evaluation study. *Neuroradiology*, doi:10.1007/s00234-018-2121-2 (2018).

16 Helmert, U. *Soziale Ungleichheit und Krankheitsrisiken*. (MaroVerl., 2003).

17 Holle, R., Happich, M., Löwel, H. & Wichmann, H. KORA--a research platform for population based health research. *Gesundheitswesen (Bundesverband der Arzte des Offentlichen Gesundheitsdienstes (Germany))* **67**, S19-25 (2005).

18 Rathmann, W. *et al.* Sex differences in the associations of socioeconomic status with undiagnosed diabetes mellitus and impaired glucose tolerance in the elderly population: the KORA Survey 2000. *The European Journal of Public Health* **15**, 627-633 (2005).

19 Rabel, M., Meisinger, C., Peters, A., Holle, R. & Laxy, M. The longitudinal association between change in physical activity, weight, and health-related quality of life: Results from the population-based KORA S4/F4/FF4 cohort study. *PLOS ONE* **12**, e0185205, doi:10.1371/journal.pone.0185205 (2017).

20 Teuner, C. M. *et al.* Impact of BMI and BMI change on future drug expenditures in adults: results from the MONICA/KORA cohort study. *BMC Health Services Research* **13**, 424, doi:10.1186/1472-6963-13-424 (2013).

21 Helbig, A. K. *et al.* Relationship between sleep disturbances and multimorbidity among community-dwelling men and women aged 65–93 years: results from the KORA Age Study. *Sleep Medicine* **33**, 151-159, doi:https://doi.org/10.1016/j.sleep.2017.01.016 (2017).

22 Inker, L. A. *et al.* Estimating glomerular filtration rate from serum creatinine and cystatin C. *New England Journal of Medicine* **367**, 20-29 (2012).
